# The rehabilitation including structured active play (RePlay) model: A conceptual model for organizing physical rehabilitation sessions based on structured active play for preschoolers with cancer

**DOI:** 10.3389/fped.2022.980257

**Published:** 2022-09-27

**Authors:** Anna Pouplier, Hanne Baekgaard Larsen, Jan Christensen, Peter Schmidt-Andersen, Helle Winther, Martin Kaj Fridh

**Affiliations:** ^1^Department of Pediatric and Adolescent Medicine, Juliane Marie Centre, Copenhagen University Hospital - Rigshospitalet, Copenhagen, Denmark; ^2^Department of Clinical Medicine, Faculty of Health and Medical Sciences, University of Copenhagen, Copenhagen, Denmark; ^3^Department of Occupational Therapy and Physiotherapy, Center of Head and Orthopedic Surgery, Copenhagen University Hospital - Rigshospitalet, Copenhagen, Denmark; ^4^Department of Nutrition, Exercise and Sports, Faculty of Science, University of Copenhagen, Copenhagen, Denmark

**Keywords:** pediatric oncology, preschoolers, rehabilitation, structured active play, physical activity, gross motor skills, social skills, personal skills

## Abstract

Anti-cancer treatments, as well as cancer itself, reduce children’s cardiorespiratory fitness, muscle strength, and gross motor functions. Early rehabilitation programs, including physical activity for childhood cancer patients, can counteract these adverse effects. Previous studies of school-aged children (6–18 years old) indicate that physical activity, including aerobic and resistance training, is safe, feasible, and effective. The goal of structured physical activity rehabilitation for preschool children (1–5 years old) is to support gross motor development and opportunities to move freely in various ways. Specific rehabilitation for preschoolers diagnosed with cancer is needed to promote physical-, social-, and personal development. This paper introduces a conceptual model—The RePlay (Rehabilitation including structured active play) Model—for organizing physical rehabilitation sessions based on structured active play for preschoolers with cancer. The theory and empirically based model combine knowledge of early childhood development, play, physical activity and rehabilitation for children with cancer, and cancer treatment. With this model, we propose how to structure rehabilitation sessions, including goal-oriented, age-sensitive, fun movement activities that facilitate preschoolers to develop gross motor skills while enhancing their social and personal skills, through four core principles: (1) ritual practices, (2) reinforcement of movement through repetition, (3) development through appropriate challenge, and (4) adjusting activities to accommodate treatment-related side effects. This model holds promise for use with preschoolers diagnosed with cancer, as it is scalable and pragmatic and accounts for the children’s fluctuating physical capacity and daily wellbeing during cancer treatment.

## Introduction

The combination of anti-cancer treatments, infections, long-term bed rest, and cancer itself reduces the cardiorespiratory fitness, muscle strength, and gross motor functions (e.g., jumping, running, throwing) of children with cancer (1–4). These impairments become evident early in treatment, leading to physical inactivity and increasing the risk of delaying important gross motor development milestones in early childhood (5). Moreover, these impairments affect long-term physical health (6, 7). Thus, early initiated rehabilitation programs are warranted for children with cancer to ameliorate the physical consequences of cancer itself and of anti-cancer treatments. Accordingly, The International Pediatric Oncology Exercise Guidelines (iPOEG) recommend children with cancer to be physically active and do what they can, when they can (8). Rehabilitation programs focusing on cardiorespiratory fitness and muscle strength have been tested for children with cancer from 4 years of age (3, 9–12). Of these studies including children with cancer, the most structured exercise interventions followed well-established guidelines for aerobic- and resistance training sessions developed for healthy populations (13, 14). These sessions included 30–40 min of aerobic/endurance training followed by ∼30 min of resistance training, including one to three sets of 6–15 repetitions for major muscle groups, with progressive exercise volume over time (10, 12). Rehabilitation programs for older children and adolescents with cancer are unsuitable for preschool children, as preschoolers are motivated to be physically active because of their need to play, learn new skills, express themselves, and interact with others (15). Furthermore, health benefits are seldom a motivating factor for children with cancer (16). Instead, they underline the social aspect of physical activity as motivating (17). A new approach involving physical activity programs based on different principles and guidelines is warranted for preschoolers with cancer (age 1–5 years).

Physical activity in early childhood is positively associated with gross motor development, and there is a positive relationship between the development of gross motor skills, social cognition, language, and social interactions (18, 19). Accordingly, a rehabilitation program for preschoolers should include gross motor skills-, social-, and personal development, as these are interdependent (15, 20, 21). Children explore their capabilities, themselves, and others, through movement and play. They discover their movement possibilities, forming their confidence in movement and an “I can” mentality (22). Play is the children’s arena for engaging and interacting with the world around them. It allows children to develop physically, socially, and personally within a world they can master with creativity and imagination (15, 23). They realize the ability to make something happen, and a distinction between instinctive movement and willful movement occurs (22).

Sufficient development of gross motor skills through movement is fundamental for: (a) engaging in social and physical activity during childhood, (b) ensuring the development of more complex movement skills, and (c) participation in physical activities throughout life (18, 24, 25). A way to achieve this is for the children to participate in structured physical activity facilitated by adults (e.g., parents, healthcare professionals, exercise professionals, teachers) and in the form of structured active play (15, 26).

In this paper, we introduce a conceptual model—The RePlay (Rehabilitation including structured active play) Model—for organizing physical rehabilitation sessions based on structured active play for preschoolers with cancer.

## Development of the rehabilitation including structured active play (RePlay) model

The RePlay Model is based on knowledge of early childhood development (25–31), physical activity-based rehabilitation interventions for children with cancer during treatment (2, 3, 17, 32–36), and empirically based experience with structured active play with healthy preschoolers and their parents.

The concept of structured active play used in this paper refers specifically to instructor-led (e.g., parents, healthcare professionals, exercise professionals, teachers) sessions of goal-oriented, age-sensitive, fun movement activities that facilitate preschoolers to develop gross motor skills while enhancing their social and personal skills (20, 23, 37). Structured active play allows children to develop positive attitudes to physical activity, which is essential for: (1) gross motor development (e.g., learning to jump, run, and throw), ensuring movement possibilities and participation in physical activity, (2) social development (e.g., learning to take turns, being patient, working together) ensuring good relationships and participation in activities with others, and (3) personal development (e.g., self-esteem, realizing abilities, confidence in movement) ensuring motivation for being physically active and taking on new challenges (37, 38). To ensure all three aspects in structured active play for children with cancer, we decided on four core principles for the model: (1) ritual practices, (2) reinforcement of movement through repetition, (3) development through appropriate challenge, and (4) adjusting activities to accommodate treatment-related side effects. The principles were defined from literature about childhood development. The principles were discussed among the author group with backgrounds in human physiology, humanities and social sport sciences, physiotherapy, sociology, and several years of experience within the field of exercise and childhood oncology. In the following, the four core principles is elaborated and why they are important to the rehabilitation of preschool children with cancer. A summary of the four core principle is presented in Table 1.

**TABLE 1:** A summary of the four core principles.

A summary of four core principles				
**Principle**	**1** **Ritual practices**	**2** **Reinforcement of movement through repetition**	**3** **Development through appropriate challenge**	**4** **Adjusting activities to accommodate treatment-related side effects**

Reasoning	Creating a routine around the structured active play with a ritual creates familiarity.	Gross motor development and gross motor skills need to be sustained through repetition, also to maintain an “I can”-mentality around movement.	The child must be challenged in new activities and gross motor functions to develop new movement skills. However, the challenge must be appropriate	Anti-cancer treatment leads to substantial variation in the children’s daily physical capacity and motivation.
What it does	Familiar rituals create certainty for the children, as well as an inclusive environment in which we start and end together.	Repetition–both through activity and movement type–will reinforce movement possibilities and ensure confidence in and sustainability of movement.	Too many activities that are too simple can promote boredom, where too many activities that are too challenging become frustrating and create the “I can’t” feeling. Activities with appropriate challenges will evoke motivation and development.	Adjusting known or new activities from session to session can be necessary to ensure participation and that the challenge is still appropriate.
How it is done	Including a starting and ending ritual which is the same every session.	Performing known activities and repetition of movement patterns, including known and new gross motor skills.	Most of the main active play activities within a session should be appropriately challenging (i.e., in the zone of proximal development.)	It can be duration adjustments, but also regressing or progressing activities to a suitable motor development level.

### Ritual practices

In early childhood, rituals help to guide behavior and support development. They contribute to a predictable structure and a secure environment in everyday life (27, 28). Children with cancer and their families experience disruptions to their daily activities and routines, as the treatment is characterized by lack of continuity, and they experience constant adjustment of old routines and the establishment of new ones (39, 40). Accordingly, creating routines around physical activity and a predictable structure is important, especially for preschoolers with cancer.

Intentional rituals ease the approach to the routine of structured active play. This is achieved by including a repeated beginning and ending ritual, hereby creating the frame around the sessions and creating certainty and familiarity for the children. The rituals thus symbolize the transition into and out of the structured active play sessions. The rituals have a social (rather than physical) purpose. They are done in a circle, whereby the participants (i.e., children, parents, instructors) can all see each other and feel seen. This creates an inclusive environment in which we start and end together. A starting and ending ritual could be a song which is performed with added movements. Following the starting ritual and preceding the ending rituals should be a known activity—a familiar, active play activity. This known activity is one with which the children are familiar and that everyone can master independently. The known activity ensures that every child feels they can participate, even if functional levels vary from child to child. These activities are core elements that will ensure that physical activity becomes a routine with recognizable and repeated elements and where all sessions begin and end with a successful experience for all children.

### Reinforcement of movement through repetition

The repeated rituals and known activities create familiar routines for the children. However, repetitions also reinforce movement. Gross motor functions are developed through guidance (verbal or physical), encouragement, and practice (25, 37). Children discover movement possibilities and corporeal powers through movement. This forms confidence in moving and creates an “I can”-mentality (22). If gross motor development is not sustained, early movement failures can result, leading to physical inactivity (37). This affects the child’s self-esteem, feeling of mastery, and movement possibilities—leading to an “I can’t”-mentality (22, 25, 37). Mastery experiences are essential for our belief in our abilities and self-efficacy, and continued success reinforces this belief (29, 30). Repetition of movement patterns, including known and new gross motor skills, will reinforce movement possibilities and sustain known gross motor skills and develop new ones. Children with cancer experience changes in their physical capabilities, movement possibilities, and associated self-esteem (41). They may experience their body letting them down and consequently find themselves being unable to do what they did before cancer and what other children can do (41). The children’s changes in experiences from movement possibilities to movement impossibilities can change their “I can” to “I can’t” (22). Hence, repeated activities through routines and repeated movement patterns ensure confidence in- and sustainability of movement. This can be exemplified with the obstacle course as an activity that is repeated from session to session, where it is possible for the child to practice and advance their abilities on the course. It can also be e repeated movement pattern like throwing several balls again and again in the same activity. When an activity or movement pattern is repeated it creates an opportunity for the child to gain continued successes in a known activity.

### Development through appropriate challenge

**FIGURE 1:**
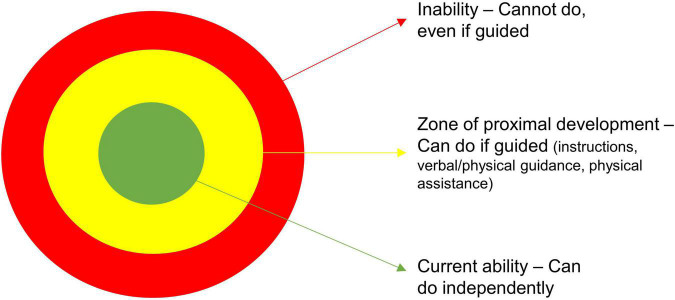
Zone of proximal development. This figure, including the colors, is based on already existing interpretations of the theory “Zone of proximal development” (31, 43, 44). In the zone of proximal development, there is a spectrum of level of guidance needed for the child to be able to do the activity (i.e., instructions, verbal/physical guidance, and physical assistance).

Repetition and mastery experiences are closely linked to the third principle of development through appropriate challenge. Appropriately challenging a child’s current level of gross motor function elicits physical adaptations, also known as progressive overload (42). The child must be challenged in new activities and gross motor functions to develop new movement skills and avoid monotonous activities, leading to demotivation. If the current level of gross motor function is not disrupted by progressive overload, adaptations will not occur (42). Appropriate challenges require encouragement and guidance to support the child in mastering the challenge (25, 29–31, 37). “Appropriate challenge” refers to Lev Vygotsky’s concept of the zone of proximal development (31). Figure 1 depicts a model based on already existing interpretations of the zone of proximal development (31, 43, 44). Too many activities that are too simple and already within mastery (green zone) can promote boredom. In contrast, excessively complex and challenging activities become frustrating and create the “I can’t” feeling (red zone). Ultimately, this leads to mastery inexperience and no development of new skills (31). Activities with appropriate challenges will evoke motivation and development. An appropriate challenge is placed in the zone of proximal development (yellow zone), where the activity or skill is too complex for the child to master independently but manageable with guidance from a knowledgeable other (i.e., parent, healthcare professional, or peer) (31).

In the zone of proximal development, there is a spectrum of how much guidance is needed for the child to master the activity: instructions, verbal/physical guidance, or physical assistance. The social interactions are essential to learn new gross motor skills, as participating in the same activities and seeing others participate in the same activity can increase the feeling of mastery and an “if they can do it, I can do it”-mentality (22, 29, 30).

Main active play activities are the core of structured active play sessions. Here, the children’s gross motor functions are challenged to either sustain or develop new movement skills. Main active play activities with repetition and guidance support the child to master experiences in known activities and new challenges. It is possible to adjust a known activity to challenge the child further, but still be within the zone of proximal development and with a sense of mastery through familiarity. For example, the child might have mastered an obstacle course with low obstacles. To challenge the child further an adjustment of the known activity is done by adding a balancing beam lifted from the ground. However, the child might now require instructions or physical guidance by showing the child how to balance over the beam, or physical assistance by holding the child’s hand. In activities within the zone of proximal development with more than one child, the relative challenge can vary from child to child in the same activity; where one child needs instructions, another needs physical guidance. However, all children can participate with different level of guidance. Furthermore, the social interactions reinforce social development, as the knowledgeable peers must show patience, wait their turn, or even offer their support to children requiring guidance.

### Adjusting activities to accommodate treatment-related side effects

The fourth principle is fundamental in childhood oncology. Anti-cancer treatment leads to substantial variation in the children’s daily physical capacity and motivation. Corticosteroids are commonly used to treat several childhood cancers—causing an altered body composition (i.e., increased fat mass, myopathy and reduced muscle mass), reduced muscle strength and adverse psychological side effects (45–47), which affects the children’s motivation and possibility to engage in structured active play. Moreover, the children can also experience dizziness, fatigue, and nausea affecting their daily physical capacity (48). This variation in physical capacity is also associated with severe neuropathic pain and peripheral neurotoxicity (i.e., a burning or prickling sensation in the peripheral limbs, sensory loss, numbness, muscle weakness, and pain) caused by chemotherapeutic agents, such as vincristine (49, 50). The severity of these symptoms can vary within hours, and they often limit the children’s physical capabilities and motivation daily. Thus, daily considerations of safety parameters (i.e., hemoglobin >5.0 mmol/l; platelets >10 billion/l at moderate to intense activities; no active diarrhea, coughing, or cold; temperature <38.0°C; no severe comorbidities) along with physician clearing the patient for physical activity, to ensure that the children can participate in exercise and to ensure to proper adjustments are warranted. Adjusting known or new activities can then be necessary from session to session (and even within sessions) to ensure that the challenge remains within the zone of proximal development. Additionally, adjusting the duration of the entire session and/or the duration or number of main active play activities can become necessary. Consequently, the length of time of the active play session and main active play activities are proportional to the daily wellbeing of the participating children. Due to the nature of cancer treatment, this means regressing activities to a lower (but suitable) motor development level or level of energy expenditure in periods with restricting side effects and progressing activities in periods with fewer side effects. Practically, this is done by: (1) decreasing/increasing the demands of the activity (fewer/more limbs involved, execution with decreased/increased range of motion, or more extensive/minor base of support), (2) use of/no use of external support (e.g., from parents, instructor), (3) use of/no use of a more static execution of activities, and (4) decrease/increase of time spent in the structured active play activities.

For example, a child who is already familiar with the obstacle course as an activity but experiences an acute worsening of their physical capacity in the following session can still obtain a feeling of mastery and the will to complete a course. This can be done by adjusting the activity with: (a) more accessible (e.g., smaller, less movable) elements, (b) added assistance from parents, such as holding their hands, or (c) both. With these adjustments, the child can participate in the same activity and maintain the “I can”-mentality.

## The rehabilitation including structured active play (RePlay) model

**FIGURE 2:**
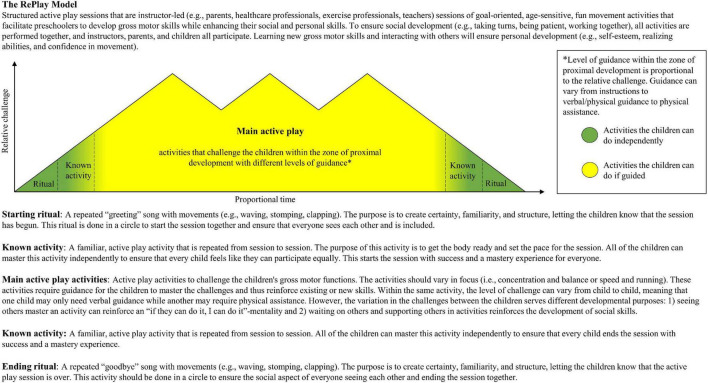
The RePlay model.

With The RePlay Model (Figure 2), we propose how to structure rehabilitation sessions, including goal-oriented, age-sensitive, fun movement activities that facilitate preschoolers to develop gross motor skills while enhancing their social and personal skills through the four core principles described above: (1) ritual practices, (2) reinforcement of movement through repetition, (3) development through appropriate challenge, and (4) adjusting activities to accommodate treatment-related side effects. The model was designed to depict how the level of challenge is relative, and the time used in each section of the session is proportional and can vary from day to day, depending on the child’s daily physical capacity and wellbeing. In Supplementary File 1, a version of the model can be found with practical examples of activities.

## Discussion and future directions

The RePlay Model was developed to provide a framework to organize a rehabilitation session aimed at supporting gross motor-, social-, and personal development in preschool children with cancer (aged 1–5 years) during treatment. These children experience impaired gross motor function (1, 4) during treatment and are at risk of delaying or missing important developmental milestones in early childhood (5). Consequently, they require early initiated and continuous rehabilitation throughout their treatment trajectory. Following the principles of disrupting the child’s current level of gross motor function through appropriate challenges and progressive overload (i.e., to elicit physical adaptations and gross motor function development) (42) is complex due to the side effects connected to the toxic nature of anti-cancer treatment. Safety parameters must be verified daily to determine participation in the rehabilitation sessions and activities must be adjusted to the child’s fluctuating wellbeing. Instead of viewing appropriate challenge of activities as a linear progress, we present a model that considers adjustments of activities to the child’s daily physical capabilities. The RePlay Model can be carried out by adults who have received instruction in it. The model is also applicable for children with all levels of gross motor function, for planning both individual- and group sessions, and can be integrated into most settings (e.g., hospital settings, home settings, school settings, outdoor settings). However, the model is not designed to monitor feasibility, adherence, and progression to physical activity interventions. Development of gross motor skills is central for preschool children and occurs through play. Consequently, well-established approaches for monitoring feasibility, adherence, and progression in physical activity interventions (e.g., heart rate, repetitions, resistance, rate of perceived exhaustion) is not suitable. We propose that registering time spent in each structured active play session, time spent in each activity, and percentage of overall time spent in main active play activities are suitable outcomes for evaluating feasibility and adherence within The RePlay Model. It is recommended that younger children (aged 1–5 years) are physically active at least 60 min each day, but preferably up to several hours (15, 51). Of this, 30–60 min should be structured physical activity facilitated by an adult (15). This also applies for children with cancer, however, iPOEG states that day-to-day differences must be taken into account and children should move when they can, as much as they can (8, 51). We propose a suitable timeframe for the model is between the 30 and 60 min with 75% of the time spent in main active play activities where the children are appropriately challenged on their current level. Progression can be monitored by registering and describing the change in the complexity of the main active play activities. This can be done by differentiating the activities in categories of levels of gross motor skills where activities are color-coded according to the child’s achieved gross motor skill level (34, 52, 53). This description can help define the intensity and difficulty of each section of the session which cannot be described with usual parameters used for older children, adolescents, or adults. The RePlay model then might be used to track progression in an intervention as an addition to the validated gross motor assessment tools.

Children develop while they play, and all children want to play, even when undergoing anti-cancer treatment. An observational study showed that preschool children with cancer still play when they are negatively affected by anti-cancer treatment, but unstructured play becomes sedentary with the increasing severity of side effects (54). Thus, families of preschool children with cancer need support to engage in physical activity as structured active play during treatment to maintain gross motor-, social, and personal development. With preschool children, structured physical activity can be challenging. The RePlay Model is based on the world of the children and their approach to movement. Here, play, social interactions, rituals, repetition, and appropriate challenges are key components to being physically active. This approach helps to keep the children motivated to engage in active play. It is unlikely that you can motivate every child with the same play activities every session, as children are curious and driven by curiosity and imagination (23, 26). The possibility to improvise is therefore necessary within a structured frame to maintain motivation. To achieve this flexibility, however, researchers risk reducing the reproducibility of a physical activity intervention. In our opinion, The RePlay Model provides a structured framework for organizing and describing rehabilitation for preschool children with cancer in a reproducible manner, but still with the flexibility needed for this patient group. However, this remains to be investigated.

Already known structured exercise programs for children with cancer are designed using progressive exercise regimes including both aerobic- and resistance exercises (3, 9, 10, 12). In most studies of exercise programs for children and adolescents with cancer, the researchers modify the exercises according to the physical capacity of the child based on daily variability (3, 9, 10, 12), showing how, despite the intent, exercise with children and adolescents with cancer is difficult to structure similarly for each session. The RePlay Model therefore gives healthcare professionals and parents a structure for organizing a rehabilitation session that can help to sustain and stimulate the child’s gross motor-, social- and personal development, even during hospitalization and active anti-cancer treatment.

The fundamental principles of The RePlay Model can theoretically be transferred to other groups of children, as play, rituals, repetition, and appropriate challenges are principles of general early childhood development. This also includes settings where gross motor, social, and personal development are the target in general (e.g., daycare, physical education classes). However, future research is needed to investigate the feasibility in these settings.

## Data availability statement

The original contributions presented in this study are included in the article/Supplementary material, further inquiries can be directed to the corresponding author.

## Author contributions

AP, HW, and MKF drafted the model and the manuscript. All authors actively and substantially contributed to the final design of the model, contributed, read, and approved the final version.
