# Supplementary figures and images for: The rehabilitation including structured active play (RePlay) model: A conceptual model for organizing physical rehabilitation sessions based on structured active play for preschoolers with cancer
Source: Front Pediatr. 2022 Sep 27;10:980257. doi: 10.3389/fped.2022.980257 (PMC9551994; doi:10.3389/fped.2022.980257)

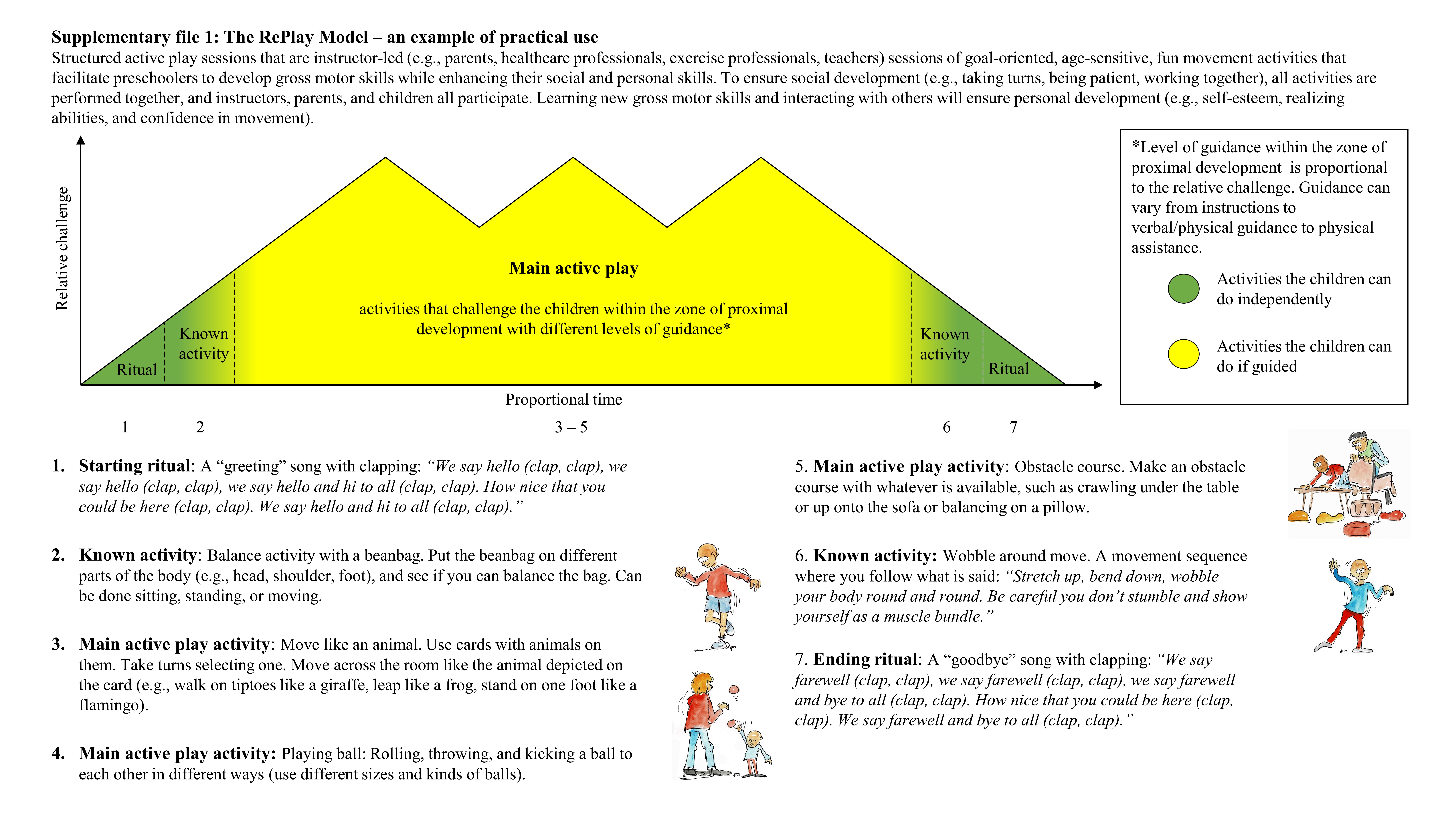

Supplement: Supplementary file 1 [file Image_1.jpg]
